# Observance de la deuxième et de la troisième dose de la chimioprévention du paludisme saisonnier chez les enfants de 3 à 59 mois et 6-9 ans dans la commune de Guidimouni, Niger

**DOI:** 10.11604/pamj.2024.49.66.45130

**Published:** 2024-11-07

**Authors:** Almoustapha Mahamane Wazodan, Mahaman Moustapha lamine, Mahamadou Doutchi, Lawali Ali Ismael, Ibrahim Alkassoum, Léon Blaise Gwendé Savadogo, Eric Adehossi

**Affiliations:** 1 Ecole Doctorale de Science de la Santé, Université Nazi Boni, Bobo-Dioulasso, Burkina Faso,; 2 Faculté des Sciences de la Santé, Centre de Formation et de Recherche en Médecine Tropicale, Université Abdou Moumouni, Niamey, Niger,; 3 Département des Sciences Biologiques, Faculté des Sciences et Techniques, Lab-EGB2S, Université André Salifou Zinder, Zinder, Niger,; 4 Département de Médecine et Spécialités Médicales, Faculté des Sciences de la Santé, Université André Salifou Zinder, Zinder, Niger,; 5 Département de la Santé Publique, Institut Supérieur des Sciences de la Santé, Université Nazi Boni, Bobo-Dioulasso, Burkina Faso

**Keywords:** Chimioprévention du paludisme saisonnier, observance, Guidimouni, Niger, Seasonal malaria chemoprevention, adherence, Guidimouni, Niger

## Abstract

**Introduction:**

la chimioprévention du paludisme saisonnier (CSP) est une stratégie efficace pour prévenir le paludisme. En 2022, le programme national de lutte contre le paludisme du Niger a décidé de faire l'extension de la CPS aux enfants de 6 à 9 ans et à cinq cycles. L'objectif de l'étude était de déterminer les facteurs influençant l'observance des deuxièmes et troisièmes doses de la CPS dans le contexte de son extension à Guidimouni, Zinder.

**Méthodes:**

des groupes de discussions ont été organisés avec des mères gardiennes d'enfants; des relais communautaires et des entretiens approfondis ont été menés avec des soignants et des autorités administratives et coutumières. Les entretiens ont été enregistrés et transcrits, ceux dans les langues locales traduits en français et les transcriptions analysées à l'aide du logiciel N'Vivo.

**Résultats:**

au total, neuf groupes de discussion et six entretiens individuels ont été réalisés. La CPS avec extension était largement acceptée comme une mesure préventive clé. L'observance de la CPS chez les parents des enfants de 3 à 5 ans et 6-9 ans semble être généralement bonne, ce qui signifie qu'ils sont en général conformes à la prise de la 2^e^ et de la 3^e^ dose. Les facteurs clés influençant l'observance sont: la mauvaise application des consignes d'administration, l'utilisation des médicaments de la CPS pour traiter d'autres maladies, la réserve d'une partie importante du médicament, ce qui peut affecter la dose seuil à la prévention et la perception de la CPS comme un traitement curatif plutôt que préventif du paludisme. La prévalence du paludisme chez les enfants de moins de 5 ans et les enfants ayant l'âge supérieur à 10 ans sont respectivement 32,03% et 35,68 en 2019; 46,76% et 37,11% en 2020, 53,07% et 49,09% en 2021, 51,93% et 45,92 en 2022. L'évolution des cas du paludisme dans cette tranche d'âge dans le village de Guidimouni ne fait qu'augmenter et présente une prévalence importante à l'égard de cette communauté malgré la chimioprévention du paludisme saisonnier.

**Conclusion:**

il est important de renforcer les actions de sensibilisation et d'éducation auprès des parents concernant la bonne administration de la CPS.

## Abstract (en)

**Introduction:**

seasonal malaria chemoprevention (SMC) is an effective strategy to prevent malaria. In 2022, Niger's national malaria control program expanded SMC to include children aged 6 to 9 years, increasing the regimen to five cycles. The objective of this study was to determine factors influencing adherence to the second and third doses of SMC within the context of its extension to Guidimouni, Zinder.

**Methods:**

focus groups were held with nannies; community relays and in-depth interviews were conducted with caregivers and administrative and customary authorities. Interviews were recorded and transcribed, those in local languages were translated into French and the transcriptions analyzed using N'Vivo software.

**Results:**

in total, nine focus groups and six individual interviews were conducted. SMC and its extension were widely accepted as a key preventive measure. Adherence to SMC among parents of children aged 3-5 and 6-9 years appeared to be generally good, indicating compliance with the second and third doses. The main factors influencing adherence included poor application of administration guidelines, the use of SMC drugs to treat other illnesses, withholding a significant portion of the medication, which can impact the threshold dose required for prevention, and the perception of SMC as a curative rather than preventive treatment for malaria. The prevalence of malaria among children under 5 years and those over 10 years was 32.03% and 35.68% respectively in 2019; 46.76% and 37.11% in 2020, 53.07% and 49.09% in 2021, 51.93% and 45.92% in 2022. Malaria cases within these age groups in the village of Guidimouni continued to rise, with a high prevalence in the community despite seasonal malaria chemoprevention efforts.

**Conclusion:**

it is crucial to strengthen awareness and education efforts for parents regarding the correct administration of SMC.

## Introduction

Le paludisme est une maladie parasitaire fébrile due à des protozoaires du genre *Plasmodium* transmis à l'homme par la piqure d'un moustique vecteur: l'anophèle femelle. Parasitose la plus fréquente dans le monde, elle reste un problème de santé publique majeur [1].

Dans le monde, la morbidité est estimée à 228 millions de cas cliniques et 405 000 décès attribuables en 2019. Près de 80% des cas de paludisme et 90% des cas surviennent en Afrique subsaharienne où les enfants de moins de 5 ans payent le plus lourd tribut. Selon les estimations en 2010, 80% de l'ensemble de décès attribuables au paludisme concernent cette classe d'âge, dont en Afrique, dans la sous-région du sahel, la plupart des cas de mortalité et de morbidités liées au paludisme surviennent pendant la saison des pluies qui dure généralement 3 à 4 mois. C'est ainsi qu'en 2012, l'OMS a émis une recommandation sur l'utilisation de la CPS pour prévenir le paludisme chez les enfants de moins de 5 ans dans des pays endémiques dont la transmission est saisonnière en complément aux autres interventions à savoir moustiquaires imprégnées d'insecticides à longue durée d'action (MIILDA), la pulvérisation intra habitation (PIH), le traitement préventif intermittent du paludisme pendant la grossesse (TPIg), la chimioprévention chez les voyageurs ainsi que la vaccination.

La chimioprévention du paludisme saisonnier (CPS) est définie comme «l'administration intermittente d'un traitement complet par un médicament antipaludique pendant la saison de haute transmission du paludisme pour éviter la maladie, l'objectif étant de maintenir des concentrations thérapeutiques de médicament antipaludique dans le sang pendant la période où le risque de contracter le paludisme est le plus élevé» [1]. L'administration répétée de la CPS pendant cette période chez les enfants en bonne santé sans manifestations cliniques permet de prévenir les cas cliniques et les décès dus au paludisme [2]. Pour que cette stratégie ait un impact sur la lutte contre le paludisme, il faut que la couverture soit élevée et prolongée pendant plusieurs saisons de transmission successives [3,4].

Dans les zones où la transmission du paludisme saisonnier est forte, notamment la zone subsaharienne de l'Afrique, il est recommandé d'administrer une CPS par l'association Sulfadoxine-Pyriméthamine plus Amodiaquine une fois par mois durant chaque saison de transmission à tous les enfants âgés de moins de 5 ans (3 mois à <59 mois). La CPS est administrée chaque mois, pendant 3, 4, 5 ou 6 mois selon la durée de la saison de haute transmission. Pour le mode d'administration, à chaque passage de la campagne (CPS), la première dose constituée de l'Amodiaquine associée à la Sulfadoxine-Pyriméthamine (AQ+ SP) est administrée sous prise supervisée; les 2^e^ et 3^e^ doses constituées de l'Amodiaquine (AQ) doivent être remises à la mère ou au gardien de l'enfant pour qu'il les lui donne à domicile. La CPS est administrée par des distributeurs (les relais communautaires et les personnels de santé, etc.) formés [5].

Au Niger, le Programme National de Lutte contre le paludisme (PNLP) a initié une étude pilote en 2013 au district sanitaire de Magaria avec l'appui de l'ONG Médecins Sans Frontières avant sa mise à l'échelle nationale. Les effets de la CPS sur la réduction de l'incidence du paludisme (50% des cas cliniques du paludisme pendant la saison de pluie en 2013) ont été rapportés, le taux de couverture (86,6%) et l'observance du traitement sont très élevés et les effets secondaires sont mineurs. Au vu de ces résultats, le PNLP a mis en œuvre la CPS à l'échelle nationale. La CPS demeure une stratégie probante, sa mise en œuvre a permis une réduction significative du paludisme chez la cible [6].

En effet, des analyses de données ont montré récemment que le fardeau du paludisme se répand vers les grands enfants, c'est dans cette optique que l'extension de la CPS chez les enfants de 6-9 ans a été inclue en phase pilote dans le district de Damagaram Takaya. Cependant, plusieurs zones d'ombres existent encore dans la mise en œuvre de cette intervention, notamment l'observance de la prise de la 2^e^et de la 3^e^dose (Amodiaquine, les deux comprimés d'Amodiaquine restant), la couverture de la population cible, la gestion des effets indésirables, la disponibilité des ressources humaines, l'accessibilité de certains endroits en plein hivernage, un taux d'incidence observé de plus en plus chez les grands enfants [7].

A cet effet, une attention particulière doit être accordée à l'observance de prise des trois doses, particulièrement les deuxièmes et les troisièmes doses qui constituent l'un des points faibles de la stratégie de la CPS marqué par la non acceptabilité et l'indisponibilité des parents. Au vu de tout ce qui précède l'étude des facteurs associés à l'observance de la CPS chez les enfants de 3 à 59 mois avec extension chez les enfants de 6-9 ans dans le département de Damagaram Takaya particulièrement dans la commune rurale de Guidimouni une zone de transmission modérée dans la région de Zinder au Niger a été réalisée.

## Méthodes

**Type d'étude:** il s'agissait d'une étude qualitative observationnelle, descriptive par entretien individuel et collectif direct visant à étudier l'observance de la chimioprévention du paludisme saisonnier chez les enfants âgés de 3 à 59 mois et 6-9 ans dans la commune rurale de Guidimouni. Cette étude s'est déroulée du 3 au 6 janvier 2023, et du 17 au 19 mars 2023.

**Figure 1:**
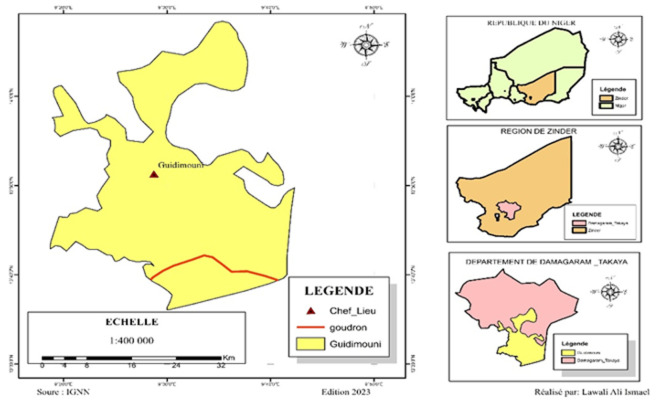
carte de la commune rurale de Guidimouni

**Site d'étude:** cette étude s'est déroulée dans le département de Damagaram Takaya particulièrement dans la commune rurale de Guidimouni, région de Zinder (Figure 1). La Commune Rurale de Guidimouni est située dans la partie Sud-Est du département de Damagaram Takaya (Figure 1). Elle couvre une superficie de 660 km^2^. Le chef-lieu de la commune est le village de Guidimouni, qui se trouve à 40 km à l'est de la ville de Mirriah et 60 km de Zinder, sur la RN1 (Axe Zinder - Diffa) (Figure 1). La population de la commune rurale de Guidimouni était de 99 447 habitants en 2022 (projection démographique INS 2012-2024 /Guidimouni 2012-2024), dont 32 482 enfants de moins de 10 ans soit 36,38%. La densité moyenne est de 128 habitants par km^2^.

**Population d'étude:** cette enquête a été réalisée auprès des parents d'enfants de 3-59 mois et de 6-9 ans ayant reçu la CPS, les autorités administratives et coutumières, les agents de santé (médecins et infirmiers), les relais communautaires impliqués dans la distribution de la CPS et le COGES.

**Critère d'inclusion et de non inclusion:** ont été inclus dans cette étude, les parents d'enfants de 3 à 59 mois et 6-9 ans qui vivent dans le district sélectionné et ayant donné un consentement éclairé, les parents qui étaient présents au passage de la CPS, et dont les enfants ont reçu la CPS. Les parents d'enfants non inclus dans la tranche d'âge, n'ayant pas reçu la CPS et n'ayant pas donné leur consentement n'étaient pas inclus dans cette étude.

**Echantillonnage et collecte des données:** nous avons constitué des focus groups de discussions et réalisé des discussions individuelles dont: Huit (8) focus groups de mères dont les enfants ont pris la deuxième et troisième dose de la CPS. Dix (10) mères/Focus; un groupe de six (6) relais communautaires ayant participé à la distribution des médicaments, des agents de santé composés du médecin CSI, le major; le COGES particulièrement le président, un entretien individuel avec les autorités administratives et coutumières dont le maire, le chef de canton, et un chef de quartier.

**Analyse des données:** nous avons utilisé la méthode d'analyse thématique. Les données qualitatives (entretiens individuels et focus group) ont été transcrites manuellement à l'aide du logiciel Microsoft Office Word 2013 et analysées par le logiciel N'VIVO 12 plus. Les données du DHIS2 ont été analysées par Excel 2013. Nous avons créé un tableau d'évaluation en utilisant les critères suivants: degré d'affinité, capacité à interagir et capacité d'influence. Une échelle de 1 à 5 était établie pour évaluer le degré d'affinité, la capacité d'interagir, la capacité d'influence où 1 représente un faible degré ou de capacité et 5 un fort degré ou de capacité.

## Résultats

Au total 92 personnes (Tableau 1) ont été interviewées dont 8 focus groups des mères gardiennes d'enfants, 1 focus group des agents de santé communautaires, le médecin du CSI, le major, le maire, le chef de canton, le chef de quartier et le président de COGES.

**Tableau 1:** taille de l'échantillon

Type de personnes	Participants	Critères d'inclusion	NBRE
**Focus group de discussion**	Mères d'enfants (10)	Les parents qui étaient présents au passage de la CPS, et dont les enfants ont reçu la CPS	8
		Accepter de participer	
	Relais communautaires (6)	Impliquer dans la distribution	1
		Acteur dans la mise en œuvre	
		Accepter de participer	
**Autorités administratives**	Maire	Acteur dans la mise en œuvre	1
		Accepter de participer	
	COGES (président)	Acteur dans la mise en œuvre	1
		Accepter de participer	
**Autorités coutumières**	Chef de canton	Acteur dans la mise en œuvre	1
		Accepter de participer	
	Chef de quartier	Acteur dans la mise en œuvre	1
		Accepter de participer	
**Agents de santé**	Médecin	Acteur dans la mise en œuvre	1
		Accepter de participer	
	Major	Acteur dans la mise en œuvre	1
		Accepter de participer	
**TOTAL**	**92 Personnes**		

### Attitude à l'égard de la CPS et son extension

***Connaissances de la communauté sur la CPS et son extension:*** la CPS est connue dans la communauté du village qui explique avec aisance l'objectif de celle-ci, comme l'expliquent certaines des participantes en ces termes:

«C'est une prévention contre le paludisme qui protège nos enfants et qui les empêcherait de manifester une forme sévère de la maladie».(FGD2_Meres).

«Ce médicament protège nos enfants contre la manifestation du paludisme…»(FGD8_Meres).

Il est à noter que les autorités coutumières ont une bonne connaissance par rapport à la CPS, comme l'expliquait certains parmi ces derniers:

«C'est un médicament utilisé pour la prévention du paludisme et l'administration procure une meilleure protection aux enfants».(Chef de Quartier).

L'extension est une stratégie bien connue par la communauté dont les renseignements sont fournis par les relais communautaires surtout lors de la distribution. Certaines participantes témoignent ceci:

«Nous avons été informé, que cette année nos enfants vont recevoir jusqu'à cinq fois ce médicament…»(FGD3_Meres).

«Vraiment cette stratégie d'extension nous arrange davantage car les enfants qui ne sont pas éligible au paravent le sont aujourd'hui».(FGD4_Meres).

***Information diffusée par les agents de santé sur la CPS et son extension:*** la sensibilisation sur la CPS et son extension notamment l'administration de la 2^e^ et de la 3^e^ dose, ainsi que les effets secondaires constituent la pierre angulaire de sensibilisation de tous les agents de santé particulièrement des relais communautaires. Les messages sont bien transmis et compris par la communauté car ils sont diffusés en langue locale et les crieurs publiques passent dans tous les lieux stratégiques afin d'atteindre le maximum des personnes. Un agent de santé nous explique la procédure:

«Avant de commencer la distribution, on prend attache avec le maire et le chef de canton pour la diffusion de l'information. Ainsi l'information est diffusée à la radio, les crieurs publiques font leur travail aussi, celui de parcourir tous les quartiers afin de diffuser l'information». (Médecin_ CSI).

Une fois sur le terrain les relais communautaires sensibilisent la communauté sur l'importance de respecter les consignes surtout les deux dernières doses et expliquent comment faire l'administration. Un relais communautaire affirme comment il expliquait l'administration aux mères d'enfants:

«Nous leur avons expliqué de façon didactique comment administrer ce médicament et nous leur expliquons de cette manière: commencer à administrer deux comprimés non semblable le 1^er^ jour puis administrer les autres comprimés restant les deux derniers jours dont un comprimé par jour».(FGD_ Relais communautaire).

La communauté avait compris le message idéal comme l'expliquait une participante au focus group N°3:

«Les distributeurs nous expliquent que quand les deux dernières doses sont respectées, cela empêcherait les enfants de manifester le paludisme dans la saison de pluie»(FGD3_Meres).

Les messages transmis ont eu des impacts positifs sur la compréhension de la communauté, une participante expliquait ceci:

«Si on doit compter sur la première dose pour avoir une prévention, alors quel serait le rôle de la 2^e^ et de la 3^e^ dose? Alors ils sont nécessaires, c'est pour cela ils sont inclus».(FGD2_Meres).

Les agents de santé ressortent que, même si la population explique les messages bien transmis et compris, les informations paraissent encore insuffisantes, et il existe un besoin de les renforcer en détail comme le notait le médecin du CSI de Guidimouni.

«Il y'a ceux qui ne donnaient pas la deuxième et la troisième dose, car même si on leur expliquait chaque jour pour que ça soit une bonne prévention, il faut les trois (3) doses, il y'a ceux qui s'entêtent pensant que leurs enfants sont bien portant pourquoi continuer à leurs donner des médicaments».(Médecin _CSI).

Il ressort dans la communauté que certains ménages considéraient que le médicament de la CPS est utilisé pour un traitement curatif. Ainsi, ils déposaient le médicament et attendaient que les enfants tombent malade. Un leader communautaire souligne cela dans ses propos: *«Elles sont nombreuses (mères ou gardiennes) celles qui n'administraient pas ce médicament et attendaient que l'enfant tombent sévèrement malade»*.(Chef de quartier).

***Acceptabilité de la CPS et son extension par la communauté:*** la CPS et son extension sont de plus en plus acceptées au sein de la communauté. Cela s'explique par leur présence à chaque moment de la distribution. Certaines participantes expliquent ceci:

«Bien sûr que oui, chaque fois nous sommes toujours disponibles pour recevoir le médicament pour nos enfants, car cela le protège contre le paludisme…» (FGD1_Meres).

«Nous sommes toujours favorables pour une nouvelle distribution. On ne sera jamais fatiguer, car il s'agit de protéger nos enfants contre le paludisme». (FGD4_Meres).

L'extension de la CPS constitue une idéologie soutenue par toute la communauté qui montre la nécessité de prolonger celle-ci, car les grands enfants font aussi la maladie, une mère gardienne confirme cela dans ces propos:

«On veut que les autorités augmentent le nombre de mois de distribution au-delà de 5 mois et aussi la fourchette d'âge soit au-delà de 9 ans…» (FGD7_Meres).

«Nous sommes très joyeuses que désormais, les enfants de 6 à 9 ans peuvent bénéficier de la CPS…» (FGD7_Meres).

Il ressort dans la communauté que certains ménages n'ont pas bénéficié de l'extension. Certaines mères d'enfants disaient que leurs enfants de plus de 5 ans n'avaient pas bénéficié de cette extension, comme l'une des participantes le prouve dans son propos:

«Ici les enfants de cette 6>-9 ans n'ont pas bénéficié de ce médicament. Nous leur (distributeurs) avons fait des reproches sur ce point. Par exemple cette fille que vous voyez, est âgée de 7 ans environs, quand ils étaient venus elle n'a pas bénéficié de ce médicament, je leur disais que le médicament est pour tous les enfants». (FGD5_Meres).

Une mère gardienne d'enfant nous expliquait cela:

«Nous avons reçu jusqu'à 4 fois ce médicament, le 3^e^ passage nous l'avons reçu lorsqu'on était au champs…» (FGD6_Meres).

Par contre d'autres expliquaient en avoir bénéficié. Une mère gardienne expliquait ceci:

«Nous avons reçu 5 passages cette année, nous avons reçu le premier passage tout au début de la saison pluvieuse et le dernier passage environs 4 mois de cela…» (FGD1_Meres).

***Qualité de collaboration entre les différents acteurs de la CPS et la communauté:*** les agents de santé et les autorités administratives entreprenaient une meilleure collaboration dans le cadre du programme de la CPS et son extension comme le prouvent certains des participants:

«En collaboration avec le service technique de la santé et les chefs traditionnels, nous essayons d'organiser ce travail dans les conditions optimales et dans l'objectif de procurer à la population le même niveau d'information.» (Maire Guidimouni).

Les chefs coutumiers particulièrement les chefs de quartiers quant à leur niveau notaient qu'ils sont marginalisés dans le processus de cette activité en tant que plus proche de la communauté comme l'expliquait un chef de quartier:

«Ils ne nous sollicitaient pas dans cette activité…Ils ne nous préviennent pas quand ils vont commencer la distribution». (Chef de quartier).

Leur implication facilitera l'accès aux ménages et permettra à la population de mieux adhérer quand ils constateront la présence d'un chef avec les distributeurs, comme l'expliquait un leader communautaire:

«Il est important d'impliquer les différents chefs des quartiers, d'appeler chacun quand son quartier est concerné pour la distribution. Une façon facile pour que la population puisse mettre davantage du sérieux quand ils constatent la présence d'un chef parmi les personnes distributeurs et ça va faciliter aux distributeurs l'accès aux ménages». (Chef de quartier).

Il ressort que la communauté entretenait une meilleure collaboration et attachait beaucoup de confiance à l'égard des distributeurs qui sont les relais communautaires comme l'expliquaient certaines participantes:

«On suit attentivement ce qu'ils nous disaient et on l'appliquait. Ils nous expliquaient comment nous allons administrer, nous sommes vraiment confiantes à eux». (FGD5_ Mères).

### Attitude et facteurs influençant l'observance de la 2^e^ et de la 3^e^ dose de la CPS et son extension

***Attitude de la communauté à l'égard de la 2e et de la 3e dose:*** l'observance de la 2^e^ et de la 3^e^ dose constitue la colonne vertébrale de la stratégie de la CPS, ne pas respecter ces deux (2) dernières doses expose à la mauvaise observance, donc le risque de développer le paludisme chez l'enfant s'intensifie. La communauté avait compris cela et exprimait de ne pas avoir trainé dans ce contexte, la CPS et son extension sont acceptées dans la communauté. Plusieurs participantes exprimaient d'être ferme dans le respect de l'administration de la 2^e^ et de la 3^e^ dose. Un extrait de certaines participantes nous dit ceci:

«Il faut obligatoirement compléter l'administration avec la 2^e^ et la 3^e^ dose pour une très bonne efficacité. On ne peut pas avoir une bonne observance en ratant les deux dernières doses, nous sommes dans l'obligation de finir toutes les plaquettes pendant les jours qui ont été consignés».(FGD4_ Mères).

On note que les mères d'enfants attachaient une confiance à l'égard des distributeurs qu'elles jugeaient bons et compétents. Elles considéraient que la CPS n'est pas un cadeau empoisonné mais plutôt bénéfique, raison de plus d'être observant à la 2^e^ et à la 3^e^ dose. Elle réduisait la dépense sanitaire et conclut que la CPS prévenait le paludisme. Elles disaient:

«Celles qui disent que le médicament peut donner des symptômes, c'est de leurs fautes car ils ne savent pas bien prendre soins de bien administrer…». (FGD1_ Mères).

Certaines recommandations émanaient des participantes qui recommandent aux autres gardiennes d'être observantes, voici ce qu'une participante disait dans ses propos:

«Nous suggérons que les mères des enfants puissent mettre le paquet dans l'administration de ce médicament à leurs enfants…». (FGD1_ Mères).

### Facteurs influençant l'observance de la 2^e^ et de la 3^e^ dose de la CPS et son extension

#### Facteurs influençant l'observance liés aux médicaments

**Niveau de connaissance de la communauté sur le médicament:** les deux médicaments utilisés dans le cadre de la CPS sont bien connus par la communauté de ce village. D'ailleurs, elle les décrit avec aisance. Une participante décrit ceci:

«Nous avons 4 médicaments alignés sur la plaquette, un comprimé de grande taille de couleur blanche et 3 comprimés de couleur jaune des petites tailles». (FGD2_ Mères).

La CPS est destinée aux enfants de 3 à 59 mois et 6-9 ans, la communauté a compris la cible à laquelle les médicaments sont destinés. Certaines mères d'enfants nous expliquaient ceci:

«Depuis des années, nous recevons ce médicament pour nos enfants, les enfants ayant l'âge de plus de 5 ans ne sont pas concerné mais cette année avec la nouvelle stratégie d'extension ils y reçoivent». (FGD5_ Mères).

«Nous leur avons fait comprendre désormais à part les enfants de moins de 5 ans, l'existence d'une extension d'âge chez les enfants de 6-9 ans…» (FGD_ Relais communautaire).

**Mode d'administration des médicaments de la CPS:** à chaque passage de la campagne (CPS), la première dose constituée de l'Amodiaquine associée à la Sulfadoxine-Pyriméthamine (AQ+ SP) est administrée sous prise supervisée; les 2^e^et 3^e^doses constituées d'Amodiaquine (AQ) doivent être remises à la mère ou au gardien de l'enfant pour effectuer l'administration. La CPS est administrée par des distributeurs (les relais communautaires et les personnels de santé) formés. Le mode d'administration de comprimés est bien connu par la population. Les mères gardiennes d'enfants connaissaient comment administrer les comprimés pendant les trois (3) jours comme l'expliquaient une des mères gardiennes:

«On administre aux enfants 1 comprimé blanc et 1 comprimé jaune le 1^er^ jour puis 1 comprimé jaune, le 2^e^ jour et un autre comprimé le 3^e^ jour. Le médicament est à administrer pour 3 jours successifs». (FGD3_ Mères)

«Le premier jour on administre un comprimé blanc avec un comprimé jaune on l'associe avec de natron et puis le deuxième jour un comprimé jaune et encore un comprimé jaune le troisième jour». (FGD4_ Mères).

Les distributeurs sont chargés à l'administration de la 1^re^ dose, mais la majorité des gardiennes expliquaient que toutes les doses sont administrées par elles-mêmes. Une mère gardienne révélait ceci:

«Ce sont les distributeurs qui administraient la 1^re^ dose, mais désormais ils n'ont plus à se fatiguer… ils nous octroyaient toutes les doses pour administrer…».(FGD6_Mères).

La communauté a compris que la 1^re^ dose n'assurait pas à elle seule une bonne prévention, elle devait nécessairement être accompagnée par les deux dernières doses comme expliquait cette participante:

«Nous savons que la première dose à elle seule ne suffit pas pour protéger nos enfants contre le paludisme…» (FGD8_ Mères).

**Figure 2:**
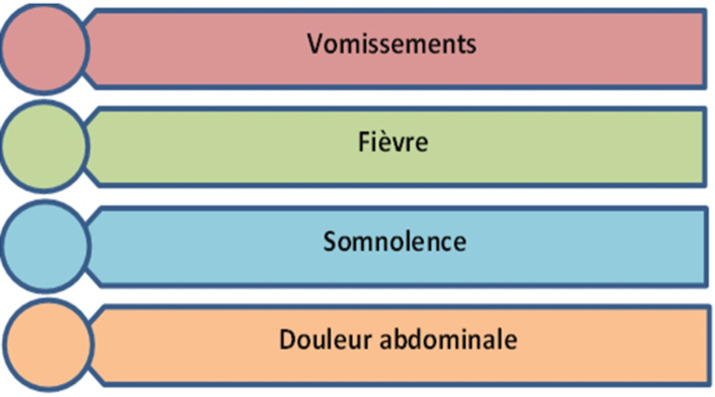
effets secondaires

**Effet secondaires liés aux médicaments de la CPS:** comme dans tout traitement médicamenteux, il existe des effets secondaires après la prise des médicaments de la CPS. Il s'agit principalement de vomissements, douleur abdominale, somnolence, fièvre (Figure 2). Les effets secondaires ne sont pas négligés dans la communauté. Une partie des participantes a mentionné ces mêmes effets suite à la prise des comprimés de la CPS chez les enfants. Certaines mères gardiennes nous témoignaient ceci:

«Nous avons vu de cas des enfants qui ont manifestés des signes comme la fièvre, le vomissement avec douleur abdominale …» (FGD8_ Mères).

Par contre d'autres mères gardiennes ont affirmé que leurs enfants ne présentaient pas des effets secondaires. Ce qui explique le fait que la survenue des effets secondaires peut varier d'un enfant à un autre, une mère gardienne nous expliquait ceci:

«Ce ne sont pas tous les enfants qui présentaient ces effets, car dans 10 enfants difficilement 2 enfants vont vomir. Sinon généralement c'est rare d'avoir ce genre d'épisode chez nos enfants». (FGD3_ Mères).

«Nos enfants ne présentaient aucun signe, le médicament est compatible avec leur organisme. Ils ne manifestent rien». (FGD4_ Mères).

Malgré la manifestation des effets secondaires (Figure 2) par quelques rares enfants, la communauté expliquait que les effets secondaires ne constituaient pas un obstacle dans l'administration de médicament, comme témoignait certaines mères gardiennes et un agent de santé:

«Ces signes ne nous empêchent pas de continuer l'administration…» (FGD4_ Mères).

«On continue l'administration jusqu'à la fin des trois (3) doses. Les vomissements, la fièvre vont disparaitre après…» (FGD8_ Mères).

### Facteurs influençant l'observance liée aux habitudes de la communauté

**Figure 3:**
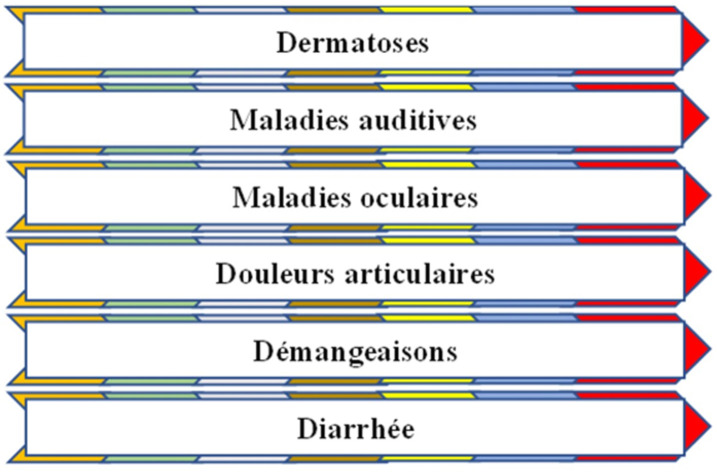
affections pathologiques

***Mauvaise utilisation de médicament de la CPS:*** il existe dans cette communauté une mauvaise utilisation à l'égard de médicament de la CPS qui est celui de son efficacité sur d'autres affections pathologiques notamment l'efficacité sur les pathologies dermatologiques (Figure 3). Plusieurs participantes ont signalé des propos dont on ne peut citer que ceux-ci:

«Nous utilisons le comprimé jaune pour traiter les plaies… Moi-même je l'ai utilisé pour ma fille et sa plait était guérie». (FGD3_ Mères).

«Nous utilisons ce médicament pour le traitement d'une maladie oculaire, on le dissout dans l'eau puis on ne l'instille goutte par goutte dans l'œil…». (FGD6_ Mères).

«Il traite la diarrhée, la fièvre, le paludisme et aussi d'autres dermatoses…». (FGD6_ Mères).

Les affections pathologiques courantes retrouvées dans cette communauté (Figure 3) incitait les mères d'enfants à la mauvaise utilisation de médicaments. Cette croyance incitait la population de ce village à réserver une grande partie de ces médicaments en cas de pathologie de ce genre. Une mère gardienne explique ceci:

«On préservait parfois quelques comprimés pour les utiliser en cas d'une dermatose».(FGD5_ Mères).

***Ignorance des consignes d'administration de la CPS:*** il est clairement expliqué que les médicaments de la CPS ne sont administrés qu'aux enfants sains, ils sont contre-indiqués pour les enfants malades. Certaines participantes utilisent ce médicament pour un traitement curatif, un leader communautaire nous expliquait ceci:

«Les mères d'enfants n'administraient les médicaments aux enfants que quand ils sont malades…» (Chef de quartier).

***Evaluation des acteurs sur la réussite de l'observance de la 2e et de la 3e dose de la CPS:*** pour évaluer les différents acteurs de la CPS nous avons émis des critères, ce sont le degré d'affinité, la capacité à interagir et la capacité d'influence (Tableau 2). Nous avons utilisé un score d'échelle de 1 à 5 où 1 indique le plus faible degré ou de capacité et 5 le plus grand degré ou de capacité.

**Tableau 2:** évaluation des acteurs sur la réussite de la prise de la 2^e^ et de la 3^e^ dose de la CPS

Critères d'acteurs	Degré d'affinité	Capacité à interagir	Capacité d'influence
Médecin	2	4	3
Major	3	4	3
Relais communautaires	4	5	5
Président de COGES	2	3	3
Maire	2	2	3
Chef de Canton	2	2	3
Chef de quartier	2	3	3
Mères d'enfants	5	5	3

**Figure 4:**
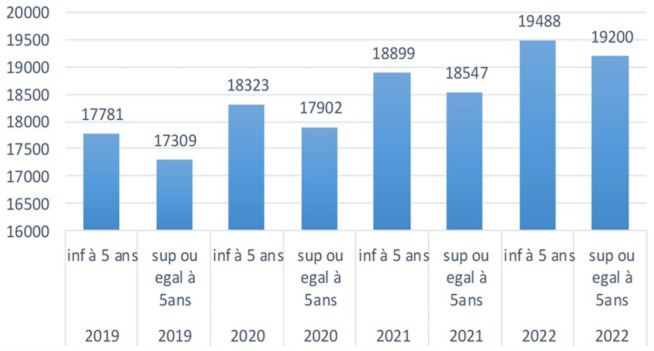
dynamique des prévalences du paludisme de 2019 à 2022 chez les enfants de moins de 5 ans et supérieur ou égal à 5 ans

**Figure 5:**
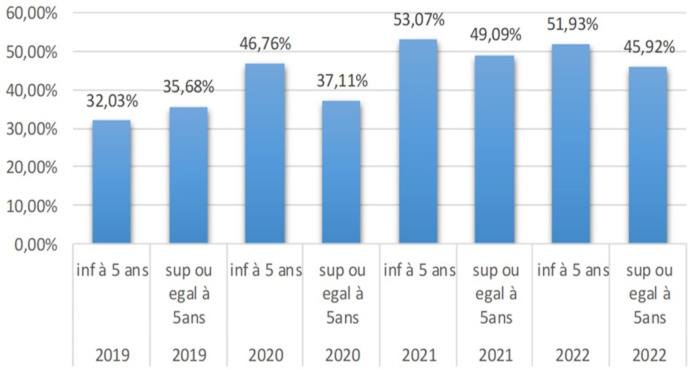
dynamique des cas du paludisme de 2019 à 2022 chez les enfants de moins de 5 ans et supérieur ou égal à 5 ans

***Dynamique des prévalences et des cas du paludisme:*** on remarque que l'évolution de cas de paludisme dans le village de Guidimouni ne fait qu'augmenter (Figure 4) et présente une prévalence importante à l'égard de cette communauté malgré la chimioprévention du paludisme saisonnier (Figure 5).

## Discussion

Les mères gardiennes d'enfants ont une bonne connaissance des messages de la CPS, leur majorité sait que la CPS est un traitement préventif, dont la distribution s'effectue en différents passages à intervalle d'un mois. Cela pourrait s'expliquer par le fait que la CPS est une activité qui est effectuée pendant des années dans la localité et aussi par l'implication des relais communautaires qui sont d'ailleurs des personnes locales faisant partie de la communauté elle-même. Il faut noter qu'au Niger, le PNLP a initié la phase pilote en 2013 au district sanitaire de Magaria qui a permis sa mise à l'échelle en 2016 [6]. Ainsi en 2016, une étude au Niger avait rapporté, que les agents de santé communautaire représentaient le principal canal de transmission de l'information sur la CPS [3]. Une étude au Nigeria avait rapporté le résultat similaire en 2023 [8].

Au total, les enfants reçoivent quatre (4) comprimés sur une période de trois jours à chaque passage. Le premier jour ils reçoivent une dose unique de Sulfadoxine/Pyriméthamine (SP) et une dose d'Amodiaquine (AQ). Les deux jours qui suivent le premier jour du traitement, les enfants reçoivent deux doses d'Amodiaquine (AQ), soit une dose par jour.

La plupart des mères gardiennes d'enfants sont bien informé et connaissent la posologie à administrer ainsi la durée du traitement de la CPS aux enfants. Mais il serait nécessaire de savoir si cette connaissance sur le mode d'administration est mise en application. En 2020, une étude au Mali avait rapporté que 98% des mères gardiennes connaissent la posologie de médicament de la CPS. En plus en 2021, une autre étude en république de Guinée avait rapporté que 96,8% connaissent la posologie de 2^e^ jour et 90,8% connaissent la posologie de 3^e^ jour ainsi 75,1% connaissent la durée du traitement en 3 jours. Cinquante virgule trois pour cent (50,3%) des mères gardiennes étaient très bien informées et 20,5% suffisamment informées [9,10]. Il faut noter que ces études n'ont pas aussi pu vérifier la mise en application de ces connaissances.

La grande majorité des mères gardiennes notaient avoir reçu et administré les médicaments de la CPS. Cela pourrait s'expliquer par la mise en œuvre de toutes les composantes de l'intervention avec l'application d'une stratégie avancée de la distribution de médicament qui explique une meilleure couverture lors de la distribution. En 2017, une étude au Burkina Faso avait rapporté qu'une interaction entre les différents facteurs modérateurs influençant le degré de mise en œuvre de la stratégie a été constatée et la mise en œuvre avait rencontré quelques difficultés en raison de la formation insuffisante des distributeurs communautaires, de l'approvisionnement insuffisant des intrants et de l'insuffisance des ressources financières. Pour la rémunération, le plaidoyer et l'encadrement mais aussi en raison de contraintes contextuelles dues à la saison des pluies [11]. En 2018, une étude en Guinée avait rapporté que les raisons les plus courantes de ne pas recevoir la CPS étaient que l'enfant était absent ou l'agent de santé communautaire n'était pas venu [12]. Une autre étude en 2021 réalisée dans huit (8) pays de l'Afrique subsaharienne avait rapporté qu'au Cameroun, au Ghana et au Nigéria, les principales raisons de ne pas fournir la CPS aux enfants étaient l'absence ou la maladie, alors qu'au Sénégal, c'était le refus parental [8].

Selon ce que rapportent les mères d'enfants, les effets secondaires de la CPS étaient mineurs chez les enfants, ce sont principalement les vomissements, la fièvre, la somnolence et la douleur abdominale. Cela pourrait s'expliquer par le fait que les manifestations des effets secondaires de tous les médicaments diffèrent d'un individu à un autre. Malgré les effets secondaires mineurs, un système de gestions de ces effets doit être mis en place pour favoriser l'adhésion des parents à la CPS. Une étude au Niger avait rapporté 27,8% des effets secondaires mineurs en 2016 dont les principales manifestations sont la diarrhée (53,7%) et le vomissement (38,8%) [3]. En 2021 au Mali, une étude avait rapporté des effets secondaires qui se manifestent régulièrement suite à la prise des médicaments dont deux principaux évènements indésirables ont été identifiés, il s'agissait de la fièvre et des vomissements [10]. Au Togo en 2018, Potchoo *et al*. avaient rapporté des cas des vomissements, de somnolence et de prurit [13]. Malgré les effets secondaires observés, les mères d'enfants questionnées notaient avoir continué l'administration de médicament.

La majorité des mères gardiennes d'enfants confirment leurs satisfactions à l'égard de la CPS car elle réduisait les épisodes palustres et les fréquentations des centres de santé. L'efficacité de la CPS à protéger les enfants contre le paludisme a été démontrée dans plusieurs études mais la mise en œuvre efficiente de la CPS reste toujours un défi majeur. En 2020, Une étude d'observation d'ACCESS-SMC avait rapporté l'efficacité de la CPS sur le paludisme en Afrique occidentale et centrale. Des études au Sénégal, au Mali et au Niger avait rapporté des résultats similaires [4,14-18].

L'extension de la CPS est une activité acceptée par la communauté, elle réduisait davantage la charge du paludisme. Cela pourrait s'expliquer par le fait que le paludisme est une maladie qui pèse sur les enfants ayant l'âge supérieur à cinq (5) ans, la mise en œuvre de l'extension de la CPS pourrait diminuer le nombre des cas de cette tranche d'âge. Des résultats similaires avaient été rapporté au Mali en 2021 et 2022 [19,20].

La majorité des parents semblent observant à la prise de la 2^e^ et de la 3^e^ dose de la CPS mais des facteurs défavorisant la bonne observance à ces deux dernières doses ont été rapportés, celui de la réserve d'une partie de médicament considérant l'utilisation médicament à titre curatif et son efficacité sur d'autres affections pathologiques. La Sulfadoxine étant un antibiotique et Pyriméthamine, un antiparasitaire, ils pourraient avoir l'efficacité sur d'autres pathologies autres que le paludisme. Une étude réalisée dans trois pays du sahel, le Niger, le Mali et le Burkina Faso avait rapporté d'autres facteurs qui sont liés à la non réception du médicament, aux cas de déplacement ou l'absence des parents, des cas de maladies, des cas de refus d'avaler le médicament et des cas de vomissement [21]. Audibert *et al*. rapportaient que les principales causes évoquées étaient l'absentéisme, la maladie des enfants, la réticence des parents et l'insuffisance de personnels [8,22].

L'analyse du tableau de critères sur l'affinité, l'interaction et l'influence sur l'observance de la deuxième et troisième dose montre que les mères d'enfants, les relais communautaires et le maire émergent comme des acteurs clés dans le réseau relationnel pour la lutte contre le paludisme saisonnier chez les enfants de 3 mois à 5 ans. Leur forte affinité, leur accessibilité et leur capacité d'influence en font des partenaires importants pour atteindre les objectifs de la CPS et notamment l'observance de la deuxième et de la troisième dose. En 2023 au Nigeria, une étude avait rapporté que l'utilisation des distributeurs communautaires de médicaments de la zone locale et l'approbation de la CPS par les leaders d'opinion locaux avait été signalée comme des facteurs clés dans l'établissement de la confiance et avait joué un rôle important dans l'acceptation et l'adoption de la CPS [22]. Les mères d'enfants ont des rôles centraux dans l'administration de la deuxième et troisième dose car à eux les relais communautaire expliquent l'administration de ces deux dernières doses donc peuvent logiquement avoir une influence majeure sur l'observance de la deuxième et troisième dose. Plusieurs études avaient noté la collaboration des mères d'enfants et les relais communautaire dans l'administration de la deuxième et troisième de la CPS, dont entre autres une étude de Diarra *et al*. au Mali en 2020, qui avait rapporté l'importance d'expliquer l'administration la deuxième et la troisième de la CPS aux mères d'enfants [23].

Le médecin, le major, le chef de quartier et le président de COGES ont des rôles centraux dans cette initiative spécifique de lutte contre le paludisme saisonnier, ils peuvent toujours contribuer de manière significative en soutenant la sensibilisation et la mise en œuvre du programme par leur connaissance de la stratégie. Selon une étude en 2020 au Mali, tous les agents de santé interrogés connaissent la posologie d'administration de SP/AQ, la survenue des effets secondaire et les contre-indications de ce médicament [24]. En 2019 au Mali, Adama Sanogo avait rapporté que la participation des personnes clés, telles que les chefs de village, les chefs religieux et d'autres chefs de groupes (jeunes ou femmes) peut impacter positivement l'adhésion de la communauté à la campagne, compte tenu de leur statut de décideurs [25].

Pour maximiser l'impact de la chimioprévention du paludisme saisonnier (l'observance de la deuxième et de la troisième dose), il serait judicieux de renforcer la collaboration avec les mères d'enfants, le relais communautaire et le maire, tout en maintenant une bonne relation avec les autres acteurs. Cette approche stratégique aidera à mobiliser efficacement les communautés et à assurer le succès de la CPS pour protéger les enfants contre le paludisme saisonnier.

La dynamique des prévalences et des cas de paludisme de 2019 à 2022 nous indique une forte prévalence et un nombre élevé des cas de paludisme malgré la CPS. Cela pourrait s'expliquer par le fait que certains enfants étaient exclus du programme de la CPS et que Guidimouni est un village qui héberge un cours d'eau permanent favorable à la prolifération des anophèles, ainsi à cela s'ajoute une variation de la pluviométrie ces dernières années. Au Niger, l'année 2020 était marquée par une augmentation des cas de paludisme suite à une pluviométrie exceptionnelle qui a occasionné des inondations, la stagnation des eaux et la prolifération des moustiques vecteurs de paludisme [26]. Selon une étude en 2018 en Côte d'Ivoire, les zones sèches jouxtant des cours d'eau semblent être favorables à la prolifération des espèces anopheliennes. Par conséquent les populations qui y vivent sont souvent très exposées aux piqures d'anophèles potentiellement infectantes [27]. Malgré les avantages apportés par la CPS dans les stratégies de lutte antipaludique, en Afrique de l'Ouest, des pays dont le Burkina Faso, le Mali et le Niger, restent fortement touchés par la maladie, avec des taux de prévalence et de mortalité élevés. Un certain nombre d'études avaient rapporté une forte prévalence du paludisme asymptomatique ou incidence élevée d'hospitalisation ou de décès en raison du paludisme dans les zones CPS. Les raisons pour lesquelles de tels résultats persistent malgré la CPS sont inconnues. Les possibilités incluent des échecs dans la couverture ou le manque d'observance des deuxièmes et/ou troisièmes doses de la CPS par les parents ou les soignants [20]. En 2018 au Mali, Doucouré avait rapporté des fortes prévalences qui ne variaient pas significativement après l'implémentation de la CPS passant de 64,8% à 58% chez les enfants de 0 à 5 ans entre 2013 à 2016 [28]. Il convient de noter que quel que soit le contexte de transmission ou le nombre de cycles, la CPS a entrainé des réductions substantielles, l'incidence et de la prévalence du paludisme, des réductions modérées du paludisme grave et de la prévalence de toute anémie chez les enfants de moins de 5 ans et chez les enfants ayant l'âge supérieur ou égal à 5 ans [29].

## Conclusion

Il ressort de cette étude que la CPS et son extension sont acceptées de plus en plus dans cette communauté. Cette étude a permis de comprendre les facteurs associés à l'observance de la CPS et de son extension ainsi que l'attitude et la perception de la communauté sur la question de la CPS et de son extension. Elle nous a permis aussi de comprendre le processus de mise en œuvre de la CPS en mettant en évidence les insuffisances à améliorer pour de meilleurs résultats. Ces insuffisances vont de la communication entre les différents acteurs à la sensibilisation des parents par les distributeurs sur l'administration de la deuxième et troisième dose chez les enfants.

### Etat des connaissances sur le sujet

- La chimioprévention du paludisme saisonnier est une stratégie de lutte contre le paludisme chez les enfants de 3 à 59 mois adapté par l'OMS depuis 2012, elle est assez bien connue et fortement appréciée par les parents des enfants;
- Les résultats des recherches antérieures ont montré des effets bénéfiques de la CPS sur la réduction de prévalence et de l'incidence du paludisme;
- D'autres études ont évalué le taux de couverture de la chimioprévention ont trouvé des résultats encourageant pour cette stratégie.

### Contribution de notre étude à la connaissance

- Face à la réussite de cette stratégie chez les enfants de moins 5 ans, il est important d'inclure les enfants de 5 à 10 ans qui ne bénéficiaient pas de la CPS et dont le taux d'incidence du paludisme est assez élevé;
- Il s'agit d'une phase pilote nos résultats pourront servir de plaidoyer pour l'extension de la CPS chez les enfants de 5 à 10 ans;
- Elle a démontré qu'il existe une insuffisance dans la communication entre les différents acteurs ; un effort de sensibilisation doit être effectué auprès des parents et des distributeurs sur l'administration de la deuxième et troisième dose chez les enfants.
